# Melanoma–Keratinocyte Crosstalk Participates in Melanoma Progression with Mechanisms Partially Overlapping with Those of Cancer-Associated Fibroblasts

**DOI:** 10.3390/ijms26167901

**Published:** 2025-08-15

**Authors:** Ramona Marrapodi, Daniela Kovacs, Emilia Migliano, Silvia Caputo, Federica Papaccio, Tiziano Pallara, Carlo Cota, Barbara Bellei

**Affiliations:** 1 Laboratory of Cutaneous Physiopathology and Integrated Center of Metabolomics Research, San Gallicano Dermatological Institute, IRCCS, 00144 Rome, Italy; ramona.marrapodi@ifo.it (R.M.); daniela.kovacs@ifo.it (D.K.); silvia.caputo@ifo.it (S.C.); federica.papaccio@ifo.it (F.P.); 2 Department of Plastic and Regenerative Surgery, San Gallicano Dermatological Institute, IRCCS, 00144 Rome, Italy; emilia.migliano@ifo.it (E.M.); tiziano.pallara@ifo.it (T.P.); 3 Genetic Research, Molecular Biology and Dermatopathology Unit, San Gallicano Dermatological Institute, IRCCS, 00144 Rome, Italy; carlo.cota@ifo.it

**Keywords:** melanoma, keratinocytes, tumour microenvironment

## Abstract

The Tumour Microenvironment (TME) is pivotal for melanoma progression and contributes to therapy resistance. While dermal cell involvement is well established, the role of epidermal cells remains less defined. To explore the contribution of Normal Human Keratinocytes (NHKs) to melanoma biology, we investigated the modification of gene and protein expression of NHKs exposed to melanoma-conditioned medium or maintained in a co-culture system. The analysis focused on pathways related to proliferation, inflammation, Extracellular Matrix (ECM) remodelling, and cell adhesion. Due to the well-documented melanoma–fibroblast crosstalk, Normal Human Fibroblasts (NHFs) and Cancer-Associated Fibroblasts (CAFs) were used as comparative references. Keratinocyte gene expression changes under the influence of melanoma secretome only partially overlapped with those of NHFs and CAFs, indicating cell-type-specific responses. Exposure to melanoma-conditioned medium induced the upregulation of bFGF, CXCL-16, TIMP-2, and E-cadherin in NHKs, alongside downregulating TGF-β and MMP-9. Although bFGF is a recognized pro-tumorigenic factor, the modulation of CXCL-16, TIMP-2, and TGF-β may reflect a protective response. Notably, under co-culture conditions, NHKs exhibited a pronounced pro-inflammatory and ECM-remodelling phenotype, characterized by elevated production of cytokines (IL-1α, IL-1β, and IL-8) and ECM-degrading enzymes (MMP-7, 9, 12, and 13), indicative of a pro-tumoral feature. Collectively, these findings underscore an active role for NHKs in melanoma initiation and progression.

## 1. Introduction

The TME is a complex dynamic ecosystem surrounding a tumour. The introduction of advanced molecular tools and high-throughput screening technologies [1,2], along with animal models [3,4], has significantly improved our understanding of the TME and its fundamental role in cancer progression. The tumour stroma is heterogeneous and comprises subpopulations of malignant and non-malignant cells, paracrine factors, and ECM components. Interactions between tumour cells and various elements of the TME promote tumour growth and invasion into adjacent healthy tissues, contributing to resistance to therapy and poor prognosis [1,5,6]. On the other hand, in the early stages of cancer development, stromal cells may exert a protective role for the host by responding to initial signals of neoplastic transformation [7]. For example, the acquisition of an environment capable of recruiting immune cells is crucial to eradicate, or at least limit, the excessive growth of precancerous cells, confining tumour to an early stage [8,9]. The many different types of cells that are part of the tissue-resident TME might facilitate the conversion into a pro-neoplastic immune microenvironment by producing various interleukins and chemokines. [10]. Studies on different types of cancer have demonstrated that the immunological composition of immune cell infiltration, particularly by cytotoxic T cells, natural killer cells, dendritic cells, and macrophages, within the TME can predict clinical outcomes and therapeutic responses [11,12,13].

The TME is an active contributor to melanoma onset, a type of skin cancer that arises from the malignant transformation of melanocytes, neural crest-derived cells that produce the pigment melanin [14,15]. In the human epidermis, fully differentiated highly dendritic melanocytes are located in the basal layer of the epidermis, surrounded by about 36 keratinocytes in a functional structure named epidermal melanin unit. To provide structural strength and protection to the skin, epidermal keratinocytes are organized into multiple layers at different stages of differentiation. The partnership with keratinocytes, ensured by tight cell–cell contact, regulates several aspects of melanocyte biology, including melanin production and distribution, and the antioxidant, anti-inflammatory, and survival capacity of melanocytes [16]. During melanomagenesis, the typical epidermal melanin unit is profoundly rearranged; however, the active role of keratinocytes in this process is still debated. Melanoma onset is a multifactorial process characterized by the interplay of genetic, environmental, and host-related determinants [17]. UV radiation exposure accounts for at least 60–70% of malignant melanoma cases [18]. UV rays exert biological effects throughout the skin [19], causing undesired reactions such as DNA damage, oxidative stress, cellular senescence, and inflammation [20,21]. As a result, chronically sun-exposed skin undergoes structural and functional changes in both dermis and epidermis [22]. These alterations may occur independently of, or precede, tumour development, creating a microenvironment conducive to carcinogenesis. Early melanocyte hyperproliferation is fully embedded in basal keratinocyte layers, which represent the major microenvironment components at this stage of the disease. Specifically, at stage 0 (melanoma in situ), cancer cells are only in the epidermis, and at stage I, the cancer cells have locally spread in the dermis. Opportunity to advance into a more aggressive disease arises from the augmented self-autonomous capacity, from external injury, and from surrounding cells. From a histological point of view, there is an enormous amount of information regarding the entire skin, including the pattern of progression, immune infiltration, the presence or not of ulceration, and the evolution into more aggressive forms [23]. Differently, from a molecular perspective, only a few studies focused on the melanoma–keratinocyte crosstalk.

Melanoma cells produce a wide range of growth factors and cytokines that support their growth and survival through autocrine signalling [24]. Conversely, they can also secrete factors that influence surrounding stromal cells and modulate the microenvironment in a paracrine manner, transforming the local tissue into a tumour-promoting habitat [25,26]. In response to paracrine signalling or direct contact with melanoma cells, fibroblasts can transform into CAFs, a heterogeneous population characterized by a persistently activated state and an enhanced ability to promote tumour growth, invasion, aggressiveness, and therapy resistance [27,28,29,30,31,32,33,34,35,36,37]. Recent studies utilizing spatial and single-cell proteomics data have investigated the characteristics and dynamics of CAFs, establishing a precise classification of different subtypes to better understand their clinical relevance [38,39,40,41].

Beyond fibroblasts, other skin cells are involved in melanocyte transformation into melanoma and disease progression. Among these, keratinocytes, the primary cellular component of the epidermis [42], are in direct contact with neoplastic cells within the melanoma niche and are likely deeply implicated in melanomagenesis, especially during the initial stages. Keratinocyte–melanocyte interactions occur through direct cell–cell contact and intense bidirectional paracrine communication. This interaction is crucial for epidermal homeostasis. Melanin, produced by melanocytes, is transferred to keratinocytes to protect them from UV-induced DNA damage. Conversely, keratinocytes stimulate melanocyte functions such as proliferation and differentiation [43]. Emerging evidence [44] suggests that this bidirectional crosstalk may evolve into a pathological interaction that promotes tumour growth, where melanoma cells influence keratinocyte behaviour, thereby fostering tumour progression. Kodet et al. demonstrated that melanoma cells contribute to significant alterations in the structural characteristics of the epidermis [45]. In human tissue samples, immunohistochemical analysis of nodular melanomas revealed a significant increase in epidermal thickness near the tumour with a highly aberrant suprabasal pattern of expression of differentiation-dependent marker keratin 14. The same study identified FGF-2, a cytokine actively produced by melanoma cells capable to control the differentiation of normal human keratinocytes, as one of the major soluble factors responsible for this phenomenon.

Desmoglein 1 (Dsg1), a protein that belongs to the cadherin family of cell adhesion molecules, is a key element for maintaining the structural integrity of tissues. Dsg1 is expressed in keratinocytes and melanocytes and is downregulated during melanomagenesis [46]. During early melanoma movement within its primary epidermal niche, melanoma cells inhibit Dsg1 protein in keratinocytes [47]. Normally, Dsg1 has a similar expression pattern to E-cadherin and is a co-receptor for E-cadherin [46]. However, Dsg1 downmodulation seems to facilitate intra-epidermal migration, independently of any detectable reduced expression of E-cadherin, showing that melanoma epidermal spread, differently to dermal invasion, is not an indirect result of loss of this cadherin.

Melanoma cells express suprabasal levels of the Notch receptor. Notch activation drives their hyperproliferative nature and transition to a more aggressive phenotype. Conversely, differentiated keratinocytes in normal skin express the Notch ligands Jagged1 and Delta-like-1 (DLL1). Thus, the shift from in situ melanoma to invasive growth is driven by a Notch-activating microenvironment in the superficial epidermis [48].

Considering the limited number of studies exploring the interaction between melanoma cells and keratinocytes in melanoma progression, we aimed to thoroughly investigate keratinocyte alterations induced by neighbouring melanoma cells. To this end, we employed primary human keratinocytes and patient-derived melanoma cells in an in vitro system.

## 2. Results

### 2.1. Preliminary Setting for the Experimental Model

Considering NHKs as the focus of our investigation, we designed the experimental model to maintain keratinocytes in their conventional experimental state, near to physiological conditions in the epidermal basal layer. Keratinocytes of the basal layer, where melanocytes reside, are highly metabolically active and are under a continuous proliferation state. In vitro, NHKs are rapidly dividing exclusively if maintained in M154 (a medium with a defined composition of mitogens lacking FBS supplementation), due to the low calcium (Ca^2+^) concentration and the presence of EGF and IGF-1 (see Section 4). Thus, to prepare the melanoma-conditioned medium to treat NHKs, we planned to shift melanoma cells into M154. to avoid melanoma culture medium which contains high calcium (Ca^2+^) concentrations capable of promoting a shift toward a less proliferative and more differentiated state [49], characteristic of the outer epidermal layers [50]. To exclude significant alterations in melanoma cell features due to the selected culture medium, we preliminarily assessed the expression of the entire gene panel selected for this study. The choice of genes relied on their known role in melanoma and TME cell interactions [17,44,51,52,53], with the aim of gaining a deeper insight into the molecular mechanisms underlying tumour progression. Specifically, the selection included growth factors, cytokines, chemokines, proteins associated with ECM remodelling, and cell adhesion proteins (Table 1).

**Figure 1:**
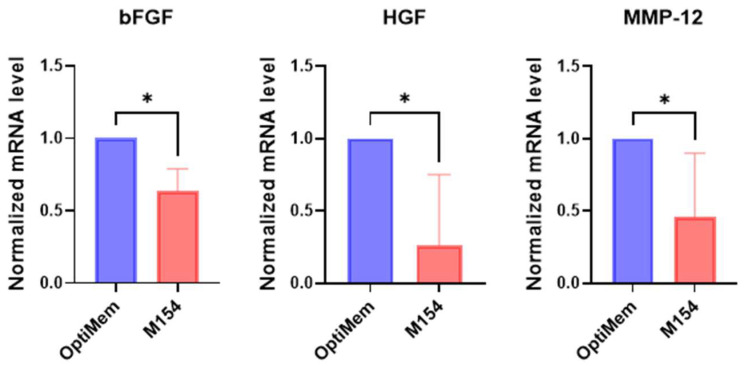
Comparative analysis of gene expression profile of melanoma cells cultured in M154 medium and OptiMem. The selected gene list was first used to evaluate the effect of M154 on six melanoma cell lines. Melanoma cells were maintained in M154 medium for 72 h prior to gene expression analysis. Melanoma cell lines were tested separately and then data were pooled for statistical analysis. Number of replicates *n* = 6. β-actin was used as the mRNA control. * *p* ≤ 0.05.

Overall, the use of M154 instead of Opti-MEM slightly altered the gene expression profile of melanoma cells. The expression of only three out of thirty investigated genes was found to be modified. However, it is noteworthy that M154, which lacks fetal bovine serum (FBS), appears to shift melanoma cells towards a less self-sufficient phenotype. This is indicated by the reduced production of bFGF and HGF (Figure 1), suggesting that cells depend on microenvironmental factors.

In agreement with the idea that in the absence of FBS, melanoma cells have limited autocrine capacity to sustain their own growth, the proliferation rate of melanoma cells was reduced in M154 compared to OptiMem containing FBS (data not included here for brevity).

Among the genes involved in tissue remodelling, a significant difference between the two culture conditions was observed only for MMP-12, a key enzyme implicated in melanoma progression.

### 2.2. Influence of Melanoma Cells on the Gene Expression Profile of NHKs

**Figure 2:**
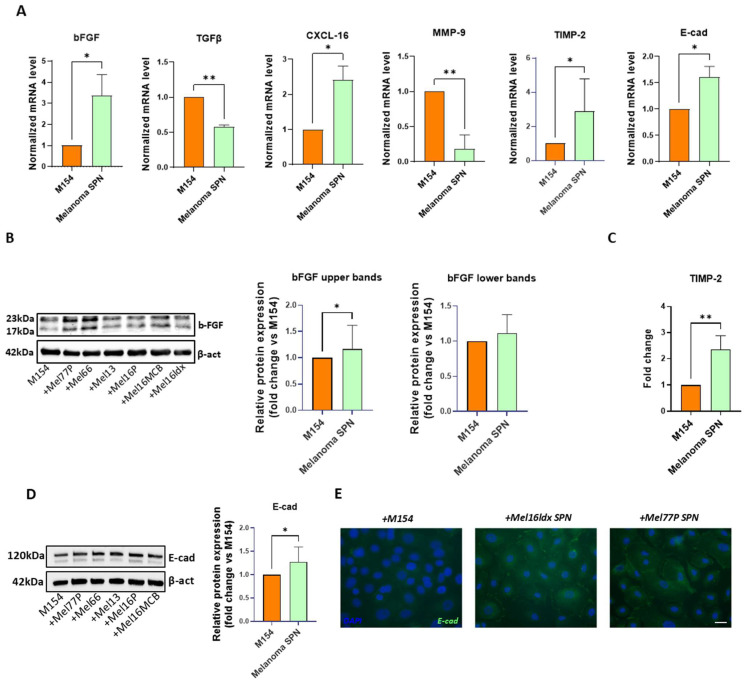
Melanoma-derived SPN significantly impacts NHK gene expression profile. Keratinocytes were treated separately with SPNs derived from different melanoma cell lines, and the results were subsequently pooled for statistical analysis. (**A**) Gene expression analysis. Comparison between NHKs after treatment with six distinct melanoma cell SPNs and control (M154). Number of replicates *n* = 6. β-actin mRNA was used as reference. (**B**) Western blot and densitometric analysis of bFGF in untreated NHKs (M154) and NHKs treated with melanoma cell SPNs. The histogram reports the densitometric analysis of the lower and upper bFGF bands. Number of replicates *n* = 6. (**C**) Quantification of TIMP-2 protein levels by ELISA, in NHKs following treatment with melanoma SPNs; the histogram reports the mean fold-change comparing untreated (M154) and treated (melanoma SPN) samples. Number of replicates *n* = 6. (**D**) Western blot and densitometric analysis of E-cadherin in untreated NHKs (M154) and NHKs treated with melanoma cell SPNs. Number of replicates *n* = 5. (**E**) Immunofluorescence of E-cadherin (green). Nuclei are counterstained with DAPI. Scale bar 20 µm. Number of replicates *n* = 2. * *p* ≤ 0.05; ** *p* ≤ 0.01.

Primary NHKs were treated with either control M154 medium or the SPN derived from melanoma cells cultured in M154 for 72 h before gene expression analysis by RT-PCR (Figure 2A). Control samples were maintained in fresh M154.

mRNA levels for bFGF, CXCL-16, TIMP-2, and E-cadherin were significantly increased. Meanwhile, the gene expression of TGF-β and MMP-9 was significantly lower than control. Interestingly, Western blot detection of bFGF (Figure 2B) revealed the presence of different isoforms, and densitometric analysis showed that the high molecular weight isoform of bFGF, frequently associated with a poor prognosis in a few human cancers [54], was significantly induced in keratinocytes by melanoma SPN. Additional confirmation at the protein level was obtained by testing the level of TIMP-2 secreted in the medium by NHKs (Figure 2C).

Attenuation of E-cadherin-mediated adhesion is considered a critical event in melanoma spreading [47]. Therefore, the observed increase in E-cadherin mRNA in keratinocytes under the influence of melanoma secretome is unexpected. To validate these results obtained by RT-PCR, we evaluated the expression level of the corresponding protein using two different techniques. Western blot analysis confirmed a significant increase in E-cadherin expression in NHKs treated with conditioned medium from melanoma cells compared to the control, providing additional support for the transcriptional findings (Figure 2D). Moreover, immunofluorescence staining demonstrated a prevalent distribution on the cell surface (Figure 2E), suggesting a full functional adhesion activity. Of interest, downregulation of E-cadherin expression in melanoma cells facilitates their detachment from keratinocytes and promotes migration into the dermis, ultimately aiding access to blood and lymphatic vessels [55,56]. Conversely, enhanced adhesion to keratinocytes via cadherins strengthens tumour–epidermis interactions, likely facilitating melanoma cells’ ability to communicate with epidermal cells.

When the data were separated into homogeneous groups distinguishing NHKs treated with SPN from primary and metastatic melanomas, all samples showed similar results regarding E-cadherin expression, although the effect of SPN from metastatic cells was stronger. However, for N-cadherin, metastatic and primary cells caused opposite results: increased expression in primary cells and decreased expression in metastatic cells. Furthermore, treatment with SPN from primary melanomas resulted in a significant decrease in TGF-β levels (Figure S1).

### 2.3. Extended Analysis of the Effect of Melanoma Cell-Derived Secretome on NHFs and CAFs

**Figure 3:**
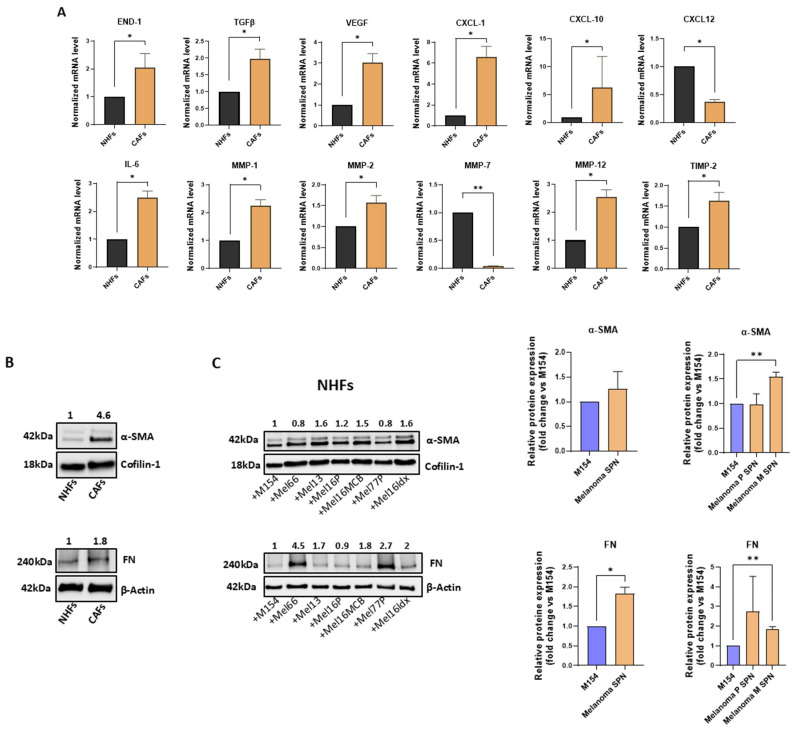
Melanoma-derived SNP considerably impacts NHF and CAF behaviour. (**A**) Gene expression analysis, comparing NHFs and CAFs cultured in M154. Number of replicates *n* = 3 for both NHFs and CAFs. Results were normalized to β-actin mRNA. (**B**) Protein detection of α-SMA and FN expression in NHFs and CAFs. Quantitative values are obtained considering untreated NHFs = 1. (**C**) Western blot and densitometric analysis of α-SMA and FN expression in NHFs both following treatment with SPNs from the six melanoma cell lines and after data stratification according to the SPNs’ derivation (primary vs. metastatic melanomas), compared to the M154 control. Quantitative values are obtained considering untreated cells = 1. Number of replicates *n* = 6. * *p* ≤ 0.05; ** *p* ≤ 0.01.

Considering the extensive studies available on the impact of melanoma cells on NHF behaviour [27,28,29,30,31,32,33,34], data obtained with NHKs were compared to a new set of experiments performed including NHFs and CAFs. First, the specific feature of CAFs was assessed comparing the gene expression profile with those of NHFs (Figure 3A).

Comparative mRNA expression analysis of the two fibroblast types revealed significantly higher expression levels of several mRNAs encoding growth factors, including END-1, TGF-β, and VEGF, inflammatory mediators such as CXCL-1, CXCL-10, and IL-6, and matrix-remodelling enzymes including MMP-1, MMP-2, MMP-12, and TIMP-2. MMP-2 plays an important role in the regulation of CAFs infiltration in melanoma [57]. Meanwhile, factors like CXCL-12 and MMP-7 were significantly lower in CAFs than NHFs. The gene expression profile observed in CAFs is indicative of their activation and of the ability to modify the TME, which is useful for melanoma progression. In addition, the intense expression of two major markers commonly used to identify CAFs, such as α-Smooth Muscle Actin (α-SMA) and Fibronectin (FN) [32,58] were assessed by the Western blot technique (Figure 3B). Interestingly, treatment of NHFs with melanoma-derived SPNs led to increased expression of α-SMA and FN compared to the control, with the upregulation of FN reaching statistical significance. Upon stratification of the data, a more pronounced and significant increase in both α-SMA and FN levels was observed in the case of SPNs derived from metastatic melanomas. α-SMA, a cytoskeletal component which gives contractile functions and elevated cellular motility to CAFs, plays a crucial role in cell movement, and its elevated expression in CAFs is often linked to poor prognosis [59,60].

**Figure 4:**
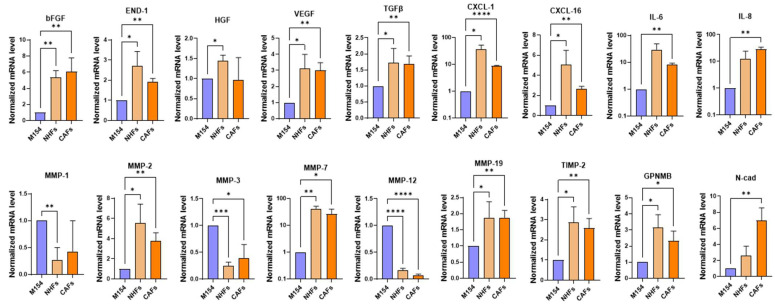
Comparative analysis of gene expression profile of NHFs and CAFs treated with melanoma-derived SPN. RT-PCR of genes of interest in NHFs and CAFs after treatment with SPN from six different melanoma cell lines. Number of replicates *n* = 6. β-actin mRNA was used as reference. * *p* ≤ 0.05; ** *p* ≤ 0.01; *** *p* ≤ 0.001; **** *p* ≤ 0.0001.

As shown in Figure 4, exposure to melanoma-derived SNP markedly impacts the gene expression of both NHFs and CAFs. Of note, eleven out of fifteen up-modulated genes (END-1, TGF-β, VEGF, CXCL-1, CXCL-16, IL-6, IL-8, MMP-2, MMP-19, TIMP-2, and N-cad) reached a higher statistical significance in the case of CAFs, demonstrating that this type of cells, in addition to having a higher basal level of expression of genes involved in melanoma progression, are more prone to address melanoma cell requirements when stimulated by their secretome. This likely reflects the pre-activation state of CAFs.

Among the genes downregulated by melanoma-derived SPN, MMP-1, MMP-3, and MMP-12 showed similar expression patterns in both NHFs and CAFs (Figure 4). Overall, comparison of the modulation of ECM factors, including MMPs and TIMP-2, revealed a pronounced effect in both NHFs and CAFs, with only minimal changes observed in NHKs (see Figure 2 and Figure 4).

Interestingly, separate analysis of data obtained from NHFs and CAFs treated with SPNs derived from primary versus metastatic melanoma cell cultures revealed a generally stronger response following treatment with SPNs from primary melanoma cells (Figure S2).

The NHF dataset demonstrated that four modified mRNAs achieved statistical significance solely when exposed to SPN collected from primary tumours (Figure S2A). Similarly, in CAF cultures, seven out of ten altered transcripts were statistically significant only in the presence of SPN derived from primary melanomas (Figure S2B). Altogether, these data argue for a minor dependence of metastatic cells compared to primary melanoma cells, that, on the contrary, firmly engage neighbouring cells. As reported in Table 2, the comparison of data obtained with NHKs, NHFs, and CAFs exposed to melanoma-derived SPNs evidenced bFGF, CXCL-16, and TIMP-2 as common features, suggesting a crucial role for melanoma biology.

### 2.4. Melanoma Cell Paracrine Network

**Figure 5:**
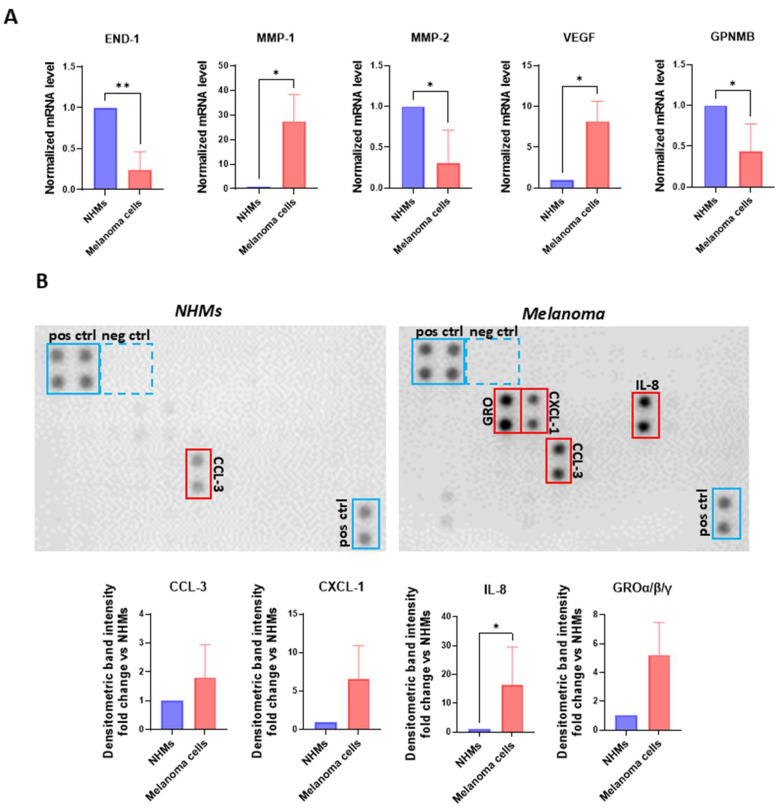
Melanoma cells release key inflammatory mediators. (**A**) Transcriptional search comparing NHMs (*n* = 1) and melanoma cells (*n* = 6) cultured in M154. β-actin was used as the mRNA control. (**B**) Protein array and densitometric analysis of human chemokines in NHMs and melanoma cells. The individual SPNs derived from the six melanoma cell lines were incubated separately with the array membranes and the results were subsequently pooled for statistical analysis. Number of replicates *n* = 6. * *p* ≤ 0.05; ** *p* ≤ 0.01.

To assess if genes regulated in NHKs and NHFs cultured under the influence of melanoma secretome are also upregulated by tumour cells, we compared the gene expression profile of melanoma cells to that of Normal Human Melanocytes (NHMs). As reported in Figure 5A, certain of the investigated genes showed the tendency of higher expression levels in melanoma cells compared to NHMs.

Notably, MMP-1 and VEGF exhibited a significant upregulation, suggesting their potential role in promoting autocrine growth, as previously reported in the literature [61]. Conversely, certain factors were downregulated in tumour cells, with END-1, MMP-2, and GPNMB showing a statistically significant reduction. An interesting observation emerged from the comparison of these data with those of Figure 4 since the relative low levels of END-1, MMP-2, and GPNMB mRNAs are likely compensated by NHFs that augmented the amount of these transcripts when stimulated by melanoma SPNs. At the same time, high level of MMP-1 expression corresponded to a reduction in the expression of this gene in both NHFs and CAFs.

To further characterize the melanoma cell secretome, individual SPNs derived from six independent melanoma cell lines underwent a semi-quantitative chemokine profiling using a human cytokine antibody array (Figure 5B). Figure 5B shows a representative array membrane comparing chemokine expression in SPNs from NHMs and melanoma cells. The membranes exhibit clear upregulation of several chemokines in the melanoma SPNs, including CCL3, members of the GRO family (CXCL1/2/3), and IL-8, all of which are highlighted in red boxes. Below the arrays, densitometric quantification is shown as fold-change relative to NHMs for each chemokine of interest. Bars represent mean values from six independent melanoma SPNs. A statistically significant increase in IL-8 was observed, while CCL3, CXCL1, and GROα/β/γ also showed elevated trends.

These factors are well known for their role in promoting inflammation, recruiting tumour-supporting immune cell subsets, and driving tumour cell proliferation, invasion, and the establishment of metastatic niches [62,63,64,65].

### 2.5. Dynamic Mutual Influence of Paracrine Activity Between NHKs and Melanoma Cells

**Figure 6:**
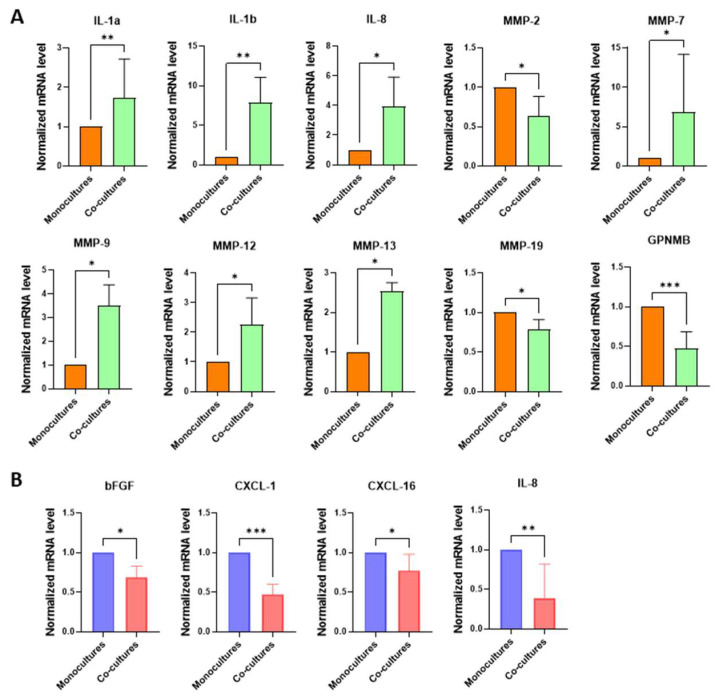
Gene expression analysis on melanoma cells and NHKs following their bidirectional crosstalk. Signature profile of (**A**) NHK and (**B**) melanoma cell co-cultures. In both cases, levels of mRNA expression measured in co-cultures were compared to monocultures, conventionally reported as 1. Number of replicates *n* = 6. β-actin was used as reference. * *p* ≤ 0.05; ** *p* ≤ 0,01; *** *p* ≤ 0.001.

To investigate the paracrine interactions between NHKs and melanoma cells more deeply, we conducted co-culture experiments using a trans-well system, which enables cells to bidirectionally communicate through their secretome without direct contact, thereby more accurately reflecting in vivo conditions. According to previous experiments, the expression of genes listed in Table 1 was evaluated in the lower compartment (NHKs) and upper compartment (melanoma cells). This analysis revealed a more pronounced pro-inflammatory signature of NHKs (IL-1α, IL-1β, and IL-8) (Figure 6A) under the dynamic influence of melanoma cells compared to samples treated only with SPNs (see Figure 2A), indicating that bidirectional crosstalk amplifies the modification of the hosting tissue by melanoma cells.

Additionally, the modulation of MMPs involved several members of this protein family, including MMP-7, MMP-9, MMP-12, and MMP-13. Unexpectedly, in the trans-well co-culture model, MMP-9 expression displayed an opposite trend compared to normal human keratinocytes (NHKs) treated with melanoma-derived soluble factors (SPNs). Notably, melanoma cells (Figure 6B) in dynamic interaction with NHKs exhibited a significant downregulation of bFGF, CXCL-1, CXCL-16, and IL-8 compared to monocultures, possibly since keratinocytes are a primary source of these factors. In particular, bFGF and CXCL-16 were markedly upregulated in NHKs exposed to SPNs (Figure 2A), whereas IL-8 showed a robust increase only in the trans-well co-culture system. These findings underscore the substantial reciprocal influence between NHKs and melanoma cells and emphasize the pivotal role of paracrine signalling in modulating cellular behaviour within the tumour microenvironment.

## 3. Discussion

In the early stages of cancer, hyperproliferative cells interact closely with resident tissue cells, which can support tumour growth before it becomes autonomous. In melanoma, the initial TME primarily comprises basal keratinocytes. Normally, melanocytes are slow-dividing cells scattered within the epidermal basal layer, maintaining close contact with 36–40 neighbouring keratinocytes, which proliferate much faster. This disparity in proliferation rates suggests continuous cycles of decoupling and recoupling within the epidermal melanin unit [66]. Disruption of this dynamic may contribute to melanoma initiation. In this context, underlying dermal fibroblasts, not in direct contact with melanocytes or melanoma cells, play a lesser role in early melanomagenesis. Progressive melanocyte proliferation disrupts epidermal architecture, leading to the loss of the epidermal melanin unit and destruction of the dermal–epidermal junction, facilitating dermal invasion and interaction with fibroblasts.

Physiologically, keratinocytes constantly renew to ensure proper turnover of differentiated cells. Maintaining a tumour-supportive epidermis requires melanoma cells to continuously modulate cell-to-cell signals, suggesting that melanoma-associated keratinocytes (MAKs) arise from sustained, intense crosstalk. Understanding changes in melanocyte–keratinocyte interactions during the transition from benign lesions to melanoma has significant clinical relevance. This differs from fibroblasts, which, as long-lived cells, retain cancer-associated fibroblast (CAF) features even after subculture in vitro [31,32]. Epigenetic mechanisms help sustain this activated CAF state independently of tumour presence. In contrast, the human epidermis renews every 40–56 days, limiting the long-term impact of epigenetic modulation on gene expression [67].

Even so, by studying molecules implicated in the initiation and progression of melanoma, we demonstrated that melanoma cell-derived SPN exerted a significant remodulation of the keratinocyte gene expression profile. The observed effect partially overlaps with those observed in the case of fibroblasts. Specifically, regarding the stimulation of bFGF, CXCL-16, and TIMP-2, both types of cells displayed a similar behaviour. bFGF is considered the most important autocrine growth factor involved in melanoma progression [25,68]. CXCL16 exhibits a multifaceted role in tumour biology, functioning as either a tumour-promoting or tumour-suppressing factor depending on the cellular and microenvironmental context. CXCL-16 promotes melanoma cell migration but does not affect proliferation or resistance to apoptosis [69]. Within the tumour microenvironment, it plays a critical role in recruiting tumour-associated cells, including cancer-associated fibroblasts (CAFs) [70]. Conversely, high CXCL-16 expression is also associated with enhanced migration and persistence of tissue-resident memory T cells, a condition that, in peripheral tissues, supports prolonged tumour immunity and progression-free survival [71], suggesting a protective role exerted by surrounding keratinocytes [72]. Consistently, elevated expression of CXCL-16 and its receptor, CXCR6, has emerged as a strong predictor of overall survival in melanoma patients [73]. TIMP-2 is a secreted protein widely expressed across various neoplastic tissues including melanoma [52]. It exhibits strong inhibitory activity against MMP-2, and its overexpression has been shown to suppress melanoma cell proliferation, partly through modulation of the Wnt/β-catenin signalling pathway [74,75]. However, other studies argued for a role in melanoma progression because TIMP-2 was found to be more elevated in cutaneous melanoma compared to nevus [76]. Recently, TIMP-2 has been implicated in the mechanism of resistance to BRAF inhibitors in melanoma cells [77]. Notably, overexpression of the multifunctional cytokine TGF-β, known to promote inflammation, angiogenesis, recruitment of cancer-associated fibroblasts (CAFs), and immune suppression, thereby supporting tumour survival and metastasis [78], was observed exclusively in fibroblasts, whereas it exhibited an opposite trend in keratinocytes. These observations further support the hypothesis that, unlike fibroblasts, keratinocytes may also play a protective role against melanoma progression.

A particularly interesting finding is the unexpected increased expression of E-cadherin in NHKs. Loss of E-cadherin frees melanoma cells from the control by keratinocytes, allows dissemination of the tumour, and is traditionally considered a marker of invasiveness in melanoma [55,56]. Notably, cadherins modulation was more pronounced following treatment with supernatants from advanced metastatic melanomas compared to those from primary melanomas, suggesting a role in tumour dissemination. In contrast, most of the other observed gene expression changes, particularly growth factors, were more marked after treatment with supernatants derived from primary melanoma cell lines, indicating a greater dependence of these cells on signals from the surrounding microenvironment. The strengthening of cadherin-mediated adhesion appears to represent a more general mechanism of interaction with surrounding cells, considering that N-cadherin expression is also induced, although significantly only in dermal cells, following treatment with melanoma-derived SPNs. On the other hand, the significance of higher E-cadherin probably needs to be explained independently of the melanocyte–/melanoma–keratinocyte adhesion function. This may reflect the previous observation that melanoma cells gain significant invasion ability following co-culture with differentiated keratinocytes [48]. Thus, it is possible that E-cadherin modulation reflects a response of NHKs attempting to maintain stronger cell adhesion, possibly reflecting the presence of a more differentiated phenotype. The role of differentiated keratinocytes in melanoma progression was further reinforced by Golan group’s study, showing that differentiated keratinocytes promote melanoma vertical invasion by activating melanoma cell Notch signalling [48]. Our data are in contradiction to the study of Mazurkiewicz and collaborators, reporting a tendency of reduced E-cadherin on HaCaT cells under the influence of a melanoma trans-well in vitro model [79]. However, HaCaT are immortalized cells maintained in high-Ca^2+^ medium, a condition that resembles the epidermal outer layers rather than the basal layer where melanocytes are physiologically located.

The most relevant difference between fibroblasts and epidermal cells consists of the ample modification of MMP family member production, in line with the idea that fibroblasts cooperate with melanoma cells in the process of dermal invasion. MMP-2, MMP-7, MMP-19, and other factors involved in tissue redesign, such as VEGF and TIMP-2, were found to be significantly up-modulated by SPNs, while the levels of MMP-1, MMP-3, and MMP-12 were markedly reduced. The gene expression signature impressed by melanoma in both NHFs and CAFs shows remarkable similarity, even if CAFs displayed at the basal level a pro-tumour distinguishing mark.

NHFs and CAFs also displayed a more pronounced pro-inflammatory profile when treated with melanoma cell secretome. Chronic inflammation in the TME may enhance melanoma cells’ ability to invade surrounding tissues and form metastatic niches, aiding in tumour dissemination. Fibroblast-derived IL-6 has been associated with both an anti-tumoral and pro-tumoral TME since it enhances the extravasation of CD8+ T cells through high endothelial venules [80]. On the other hand, IL-6 may also play an important role in shaping the immune environment by participating in the polarization of macrophages and neutrophils toward an immunosuppressive phenotype [81]. Similarly, IL-8 plays a crucial role in melanoma progression by promoting tumour cell proliferation, migration, and angiogenesis. Elevated IL-8 levels are often associated with poor prognosis and increased metastatic potential [82]. In trans-well, melanoma cells compensate the augmented IL-8 expression driven by its secretome, reducing the autocrine production. In the case of keratinocytes, a significant upregulation of interleukins (IL-1α, IL-1β, and IL-8) was demonstrated only in trans-well co-culture experiments. It seems that in some way, melanoma cells counterbalance the strong inflammatory feature of neighbouring cells to avoid a critical level of inflammation.

## 4. Materials and Methods

### 4.1. Ethics Statement

All patients gave written informed consent for collecting human specimens for research following the tenets of the Declaration of Helsinki. In addition, the Institutional Research Ethics Committee (Istituto Regina Elena e San Gallicano) approved all research activities involving human subjects.

### 4.2. Cell Cultures and Treatments

NHKs, NHFs, and NHMs were isolated from skin fragments obtained during plastic surgery. Briefly, the skin was cut into pieces of approximately 4 mm^2^ and incubated overnight at 4 °C with Dispase (2.5 mg/mL) to separate the epidermal layer from the dermis. The dermis was then enzymatically digested with 0.35% *v/v* collagenase for 1 h at 37 °C. Meanwhile, epidermal cells were extracted with trypsin in PBS (Euroclone S.p.A., Milan, Italy) for 10 min at 37 °C to obtain a single-cell suspension (NHKs and NHMs). Selective culture media were then used to isolate homogeneous cultures: medium 154 (M154) (Gibco-Thermo Fisher Scientific, Waltham, MA, USA) supplemented with Human Keratinocyte Growth Cocktail (HKGS; Cascade Biologics Inc., Portland, OR, USA), Ca_2_^++^ (0.07 mM), glutamine, and antibiotics were used for NHKs, and medium 254 (M254) (Gibco-Thermo Fisher Scientific) supplemented with Human Melanocyte Growth Cocktail (HMGS; Cascade Biologics Inc., Portland, OR, USA) in the case of NHMs. Specifically, HKGS contains bovine pituitary extract 0.2% *v*/*v*, IGF-1 0.01 μg/mL, transferrin 5 μg/mL, hydrocortisone 0.18 μg/mL, and EGF 0.2 ng/mL, whereas HMGS contains bovine pituitary extract 0.2% *v*/*v*, IGF-1 0.01 μg/mL, transferrin 5 μg/mL, bFGF, hydrocortisone 0.18 μg/mL, heparin 3 μg/mL, phorbol 12-myristate 13-acetate 10 ng/mL, and a low concentration of FBS (0.5% *v*/*v*). For NHFs, DMEM (EuroClone S.p.A.) supplemented with 10% Fetal Bovine Serum (FBS), glutamine, and antibiotics (Hyclone Laboratories, South Logan, UT, USA) was used. Melanoma cells were isolated from excess portions of surgical specimens taken for histological examination, without compromising standard diagnostic procedures. The tissue was manually fragmented into small pieces, incubated with 0.35% collagenase for 45 min at 37 °C, centrifuged, resuspended, and cultured in OptiMEM (Life Technologies, Invitrogen, Milan, Italy) containing 10% FBS and antibiotics. This study included a total of six melanoma cell lines. Specifically, as described in Table S1, three cell lines were derived from primary skin melanoma, two cell lines were derived from skin metastasis, and one additional cell line was isolated from lymph node metastasis. A small amount of the cells (about 1/10) obtained from melanoma sample digestion was grown in DMEM plus 10% FBS, glutamine, and antibiotics to isolate CAFs. The absence of tumour cells was certified by a negative signal in immunofluorescence routinely performed to detect tyrosinase and HMB45 melanoma-associated markers. All experiments used short-term cell cultures (2–12 passages for NHFs, CAFs, and melanoma cells; 1–4 passages for NHKs and MHMs). To prepare melanoma Supernatant (SPN), cells were cultured with M154 for 72 h before harvesting the conditioned medium. The SPN was centrifuged at 2500× *g* for 20 min and stored at −80 °C. Each SPN was used and measured separately. In the case of consistent gene expression differences, the data were combined to increase statistical robustness of the analysis. Whenever distinct effects were observed between primary and metastatic melanoma cell-derived media, these two groups were analyzed separately. NHKs, NHFs, and CAFs were treated with the previously prepared SPN from melanoma cells and incubated at 37 °C for 72 h before RNA and protein extraction.

To establish co-culture experiments, keratinocytes were seeded in 6-well plates where, at the same time, melanoma cell lines were seeded on 3 μm inserts. Both cell types were cultured in M154 medium. On the following day, the inserts containing melanoma cells were placed into the wells containing keratinocytes and fresh medium. Co-cultures were maintained for 72 h before mRNA extraction. Keratinocyte and melanoma cell monocultures served as controls.

### 4.3. Gene Expression Analysis

Total RNA was extracted using the Aurum Total mini kit (Bio-Rad Laboratories, Milan, Italy). cDNA was synthesized from 1 µg of total RNA using PrimeScript™ RT Master Mix (Takara Bio Inc., Beijing, China) according to the manufacturer’s instructions. Quantitative Real-Time PCR (RT-PCR) was performed by reaction mixture containing SYBR qPCR Master Mix (Vazyme Biotech Co., Ltd., Nanjing, China) and 25 pmol each of forward and reverse primers using (Bio-Rad Laboratories). All samples were analyzed in triplicate and relative expressions were determined by normalizing the results to β-actin mRNA. A preliminary set of experiments was used to evaluate β-actin expression stability in the presence of treatments. To do that, we preliminary compared the β-actin mRNA amount to other candidate housekeeping genes. Sequences of target genes and primers used are listed in the Supplementary Materials (Table S2).

### 4.4. Western Blot Analysis

Cell extracts were prepared using RIPA extraction buffer containing protease and phosphatase inhibitors. Proteins were separated on SDS-polyacrylamide gels and transferred to a nitrocellulose membrane. Then non-specific binding sites were saturated with EveryBlot blocking buffer (Bio-Rad Laboratories), and the membranes were probed with the following primary antibodies: rabbit monoclonal anti-E-cadherin (1:1000) (Antibodies.com, Stockholm, Sweden), anti-basic FGF (1:500) (Upstate cell signaling solutions, Lake Placid NY), anti-α-SMA (1:1000) (Sigma Aldrich, Merck, Milan, Italy), and anti-fibronectin (1:1000) (Sigma Aldrich, Merck). The anti-β-actin (1:10.000) (Sigma Aldrich, Merck) or anti-cofilin 1 (1:10,000) (Bio-Rad Laboratories) antibodies were used to normalize the protein content of each sample. β-actin and Cofilin-1 were alternatively used as protein loading control, depending on the molecular weight of target proteins. The secondary antibody complexes, anti-mouse or anti-rabbit conjugated with horseradish peroxidase (Cell Signaling Technology, Beverly, MA, USA), were detected by chemiluminescence. Image acquisition and densitometric analysis were performed using the ChemiDoc acquisition system (Bio-Rad Laboratories).

### 4.5. Enzyme-Linked Immunosorbent Assay (ELISA) for TIMP-2 Quantification

Keratinocyte quantification of TIMP-2 protein levels was performed using a human TIMP-2 ELISA kit (RayBiotech, Norcross, GA, USA), following the manufacturer’s guidelines. Keratinocyte conditional medium was used to test the level of TIMP-2. Absorbance was measured at 450 nm using a microplate reader (DTX 880 Multimode Detectore, Beckman Coulter, Brea, CA, USA). A standard curve was generated using known concentrations of TIMP-2, and sample values were interpolated accordingly. All assays were performed in duplicate and normalized to total protein content.

### 4.6. Chemokine Protein Array

Human chemokine expression was analyzed using a commercially available antibody array system (Human Chemokine Array C1 RayBiotech Life, Inc., Peachtree Corners, GA, USA), which uses membrane-bound chemokine-specific antibodies to capture chemokines in biological fluids. The procedure was performed according to the manufacturer’s instructions. The SPN of melanoma cells cultured in M154 was collected according to the procedure described above. Membranes were blocked with 2 mL of 1× blocking buffer for 30 min at RT and then incubated with 1 mL melanoma medium at 4 °C overnight. The SPN was then decanted from each well and the membranes were washed three times with 2 mL 1× Wash Buffer I, followed by two washes with 2 mL 1× Wash Buffer II at RT. The membranes were then incubated in biotin-conjugated primary antibodies for 2 h at RT, washed as above, and incubated in 1:1000 diluted horseradish peroxidase-conjugated streptavidin for 2 h. After a final cycle of wash, membranes were then washed thoroughly and incubated with a chemiluminescent ECL substrate for 5 min at RT. Images were captured and densitometric analysis were performed using a UVITEC Mini HD9 imaging system (Alliance UVItec Ltd., Cambridge, UK).

### 4.7. Immunofluorescence

Keratinocytes, seeded on coverslips and at the experimental end point, were fixed with 4% paraformaldehyde (PFA) for 20 min at RT followed by 0.1% Triton X-100 to allow permeabilization. The cells were then incubated with the primary antibody rabbit monoclonal anti-E-cadherin (1:200) (Antibodies.com, Stockholm, Sweden). After coverslip wash with PBS, samples were incubated with goat anti-rabbit Alexa Fluor 488-conjugate (1:800) (Cell Signaling Technology). After washing with PBS, coverslips were then mounted using ProLong Gold antifade reagent with DAPI (Invitrogen, Life Technologies Corporation, Carlsbad, CA, USA). Fluorescence signals were acquired using a CCD camera (Zeiss, Oberkochen, Germany).

### 4.8. Statistical Analysis

The results in the figures are representative of multiple experiments performed with NHKs, NHFs, and CAFs using SPN from six independent melanoma cell lines. Quantitative data are presented as a mean and standard deviation (SD) calculated using GraphPad Prism 10 software. The parametric *t*-test was used to assess statistical significance with thresholds of * *p* ≤ 0.05, ** *p* ≤ 0.01, *** *p* ≤ 0.001, and **** *p* ≤ 0.0001.

## 5. Conclusions

Our findings demonstrated a phenotypic modification of keratinocytes under the influence of melanoma secretome, suggesting a possible role within the melanoma microenvironment. Keratinocytes may contribute significantly to the early phases of neoplastic transformation by modulating tumour responses and shaping a microenvironment that supports melanoma progression. By modulating their secretome, melanoma cells actively engage in crosstalk with the surrounding tissue to establish conditions favourable to their survival and dissemination. Interestingly, some alterations observed in keratinocytes following exposure to melanoma-derived secretome also suggest the activation of protective mechanisms aimed at mitigating damage caused by tumour cells. A limitation of this study lies in the methodological approach, as the choice of keratinocyte medium favours the biology of these cells but may not be the most reliable in vitro model for melanoma cells and fibroblasts. Further in vivo investigations could help to strengthen these results.

## Figures and Tables

**Table 1:** List of different genes evaluated by RT-PCR. bFGF (Basic Fibroblast Growth Factor); END-1 (Endothelin-1); END-3 (Endothelin-3); IGF-1 (Insulin-Like Growth Factor 1); HGF (Hepatocyte Growth Factor); SCF (Stem Cell Factor); TGF-β (Transforming Growth Factor Beta); VEGF (Vascular Endothelial Growth Factor); IL-1α/1β/6/8/17 (Interleukin 1 Alpha/1 Beta/6/8/17); CXCL-1/9/10/12/16 (CXC Chemokine Ligand 1/9/10/12/16); MMP-1/2/3/7/9/12/13/19 (Matrix Metalloproteinase 1/2/3/7/9/12/13/19); TIMP-2 (Tissue Inhibitor of Metalloproteinase 2); GPNMB (Glycoprotein Non-Metastatic Melanoma Protein B).

Growth Factors	Cytokines	Chemokines	Matrix Metalloproteinases	Adhesion Proteins
bFGF, END-1, END-3, IGF-1, HGF, SCF, TGF-β, VEGF	IL-1α, IL-1β, IL-6, IL-8, IL-17	CXCL-1, CXCL-9, CXCL-10, CXCL-12, CXCL-16	MMP-1, MMP-2, MMP-3, MMP-7, MMP-9, MMP-12 MMP-13, MMP-19 TIMP-2	E-cadherin GPNMB N-cadherin

**Table 2:** mRNA targets analyzed in NHKs, NHFs, and CAFs, comparing cells in M154 with those treated with melanoma SPN. The red and light blue arrows, respectively, indicate a statistically significant increase or decrease in the target mRNA. The grey arrow indicates an increase or decrease that is not statistically significant. ND undetectable. = no difference between the control sample in M154 and melanoma cells treated with SPN. The table reports differences over untreated controls corresponding to ≥2.0 fold increase, ≤0.5 fold decrease, and other differences reaching the statistical significance ≤0.05.

Target mRNA	NHKs		NHFs		CAFs	
bFGF	3.4 ± 1.0	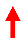	5.3 ± 0.8	** 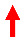 **	6 ± 1.7	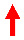
END-1	1.0 ± 0.6	=	2.7 ± 0.7	** 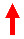 **	1.9 ± 0.2	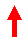
END-3	ND		ND		ND	
HGF	ND		1.4 ± 0.1	** 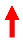 **	1 ± 0.6	=
IGF-1	2.8 ± 1.6	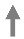	ND		ND	
SCF	0.9 ± 0.3	=	ND		ND	
TGFβ	0.5 ± 0.1	** 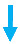 **	1.7 ± 0.4	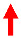	1.7 ± 0.2	** 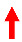 **
VEGF	0.8 ± 0.3	=	3.1 ± 0.8	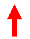	3 ± 0.5	** 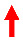 **
IL-1α	0.7 ± 0.2	=	ND		ND	
IL-1β	1.7 ± 1	=	ND		ND	
IL-6	1 ± 1.7	=	29.7 ± 18.7	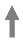	8.1 ± 1.1	** 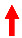 **
IL-8	0.8 ± 0.2	=	12.7 ± 11.3	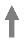	28.8 ± 5.1	** 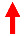 **
IL-17	ND		ND		ND	
CXCL-1	0.8 ± 0.7	=	36.1 ± 16.3	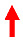	8.7 ± 0.5	** 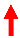 **
CXCL-9	ND		ND		ND	
CXCL-10	1.1 ± 0.6	=	ND		ND	
CXCL-12	ND		=		=	
CXCL-16	2.3 ± 0.5	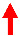	5 ± 1.4	** 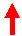 **	2.6 ± 0.3	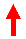
MMP-1	1.1 ± 0.6	=	0.3 ± 0.2	** 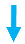 **	0.4 ± 0.6	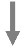
MMP-2	0.8 ± 0.3	=	5.5 ± 1.8	** 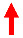 **	3.8 ± 0.8	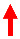
MMP-3	7.1 ± 8	** 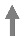 **	0.3 ± 0.06	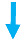	0.4 ± 0.2	** 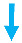 **
MMP-7	3.9 ± 3.5	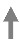	42.1 ± 9.6	** 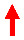 **	26.4 ± 14	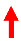
MMP-9	0.2 ± 0.1	** 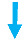 **	ND		ND	
MMP-12	0.7 ± 0.6	=	0.2 ± 0.03	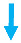	0.08 ± 0.02	** 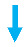 **
MMP-13	1.4 ± 0.8	=	ND		ND	
MMP-19	1.2 ± 0.3	=	1.9 ± 0.5	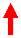	1.9 ± 0.2	** 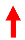 **
TIMP-2	2.8 ± 1.2	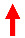	2.9 ± 0.8	** 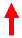 **	2.6 ± 0.5	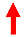
E-cadherin	1.6 ± 0.2	** 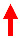 **	ND		ND	
GPNMB	4.3 ± 2.2	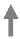	3.1 ± 0.8	** 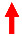 **	2.3 ± 0.6	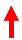
N-cadherin	0.8 ± 0.8	=	2.6 ± 1.2	** 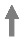 **	7 ± 1.6	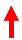

## Data Availability

Data are available on request.

## Supplementary Materials

The following supporting information can be downloaded at: https://www.mdpi.com/article/10.3390/ijms26167901/s1.
