# Supplementary material for: Melanoma–Keratinocyte Crosstalk Participates in Melanoma Progression with Mechanisms Partially Overlapping with Those of Cancer-Associated Fibroblasts
Source: Int J Mol Sci. 2025 Aug 15;26(16):7901. doi: 10.3390/ijms26167901 (PMC12386853; doi:10.3390/ijms26167901)
Supplement: Supplementary file 1 [file ijms-26-07901-s001.zip › ijms-3782709-supplementary.pdf]

Distinct effects of primary and metastatic melanoma cells on NHKs, NHFs and CAFs.

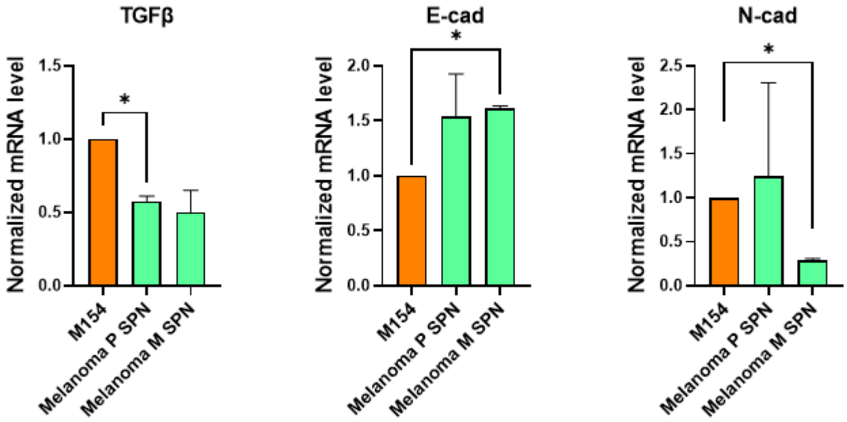

Figure S1. NHKs transcriptional profiling treated with the SPN from primary (Melanoma P SPN) and metastatic (Melanoma M SPN) melanoma cells compared to the control in M154. β-actin was used as the mRNA control. * $p \leq 0.05$.

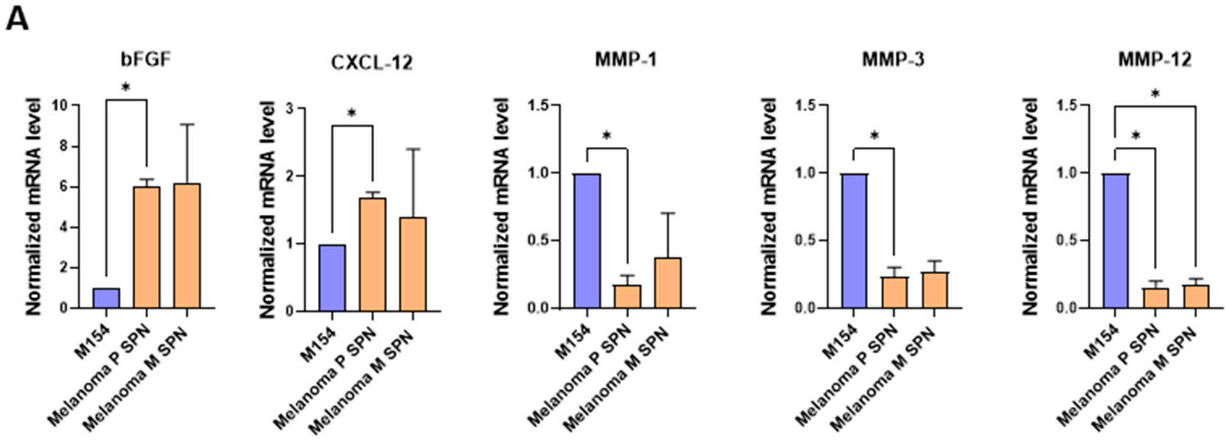

B

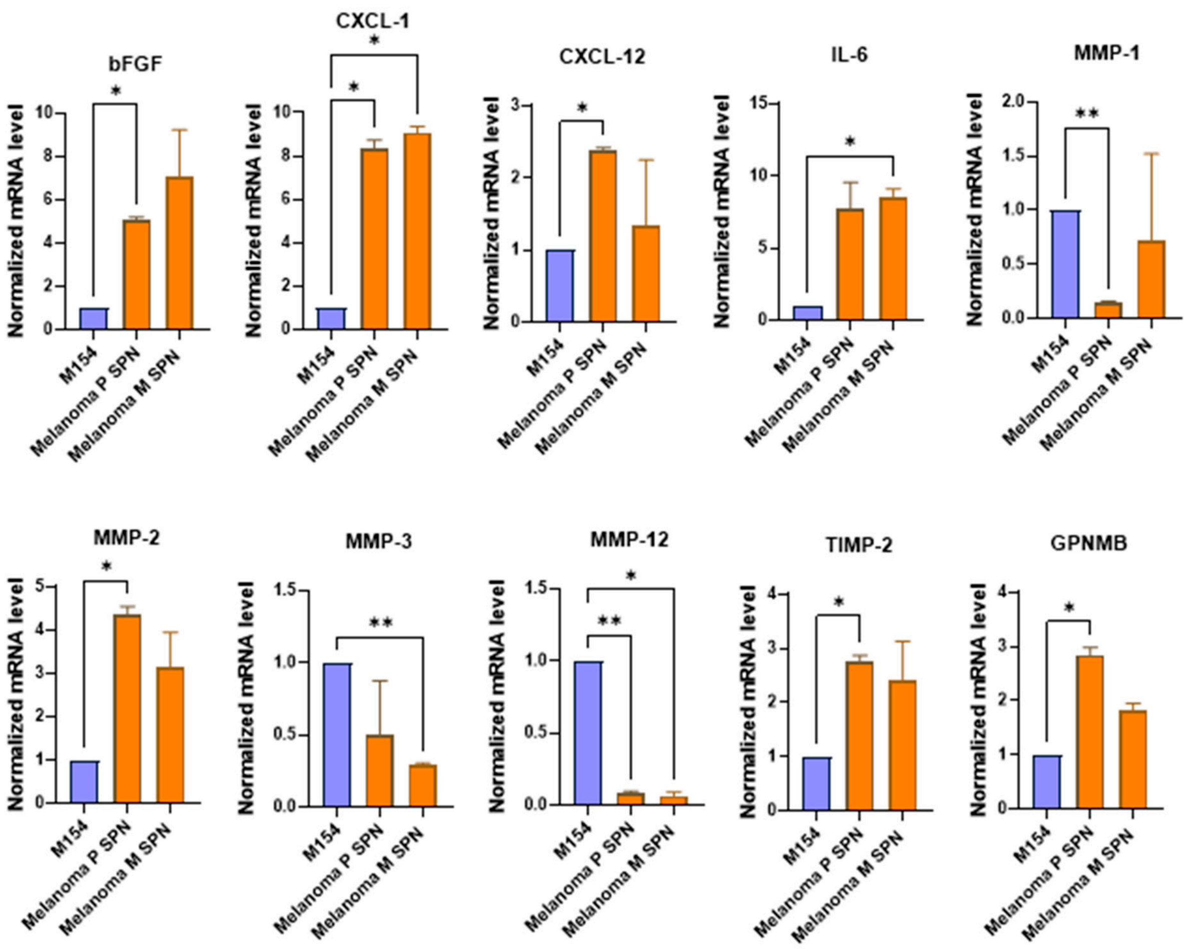

Figure S2. Gene signature of NHFs **(A)** and CAFs (**B**) treated with the SPN from primary (Melanoma P SPN) and metastatic (Melanoma M SPN) melanoma cells compared to the control in M154. β-actin was used as the mRNA control. * $p \leq 0.05$; ** p≤ 0,01**.**

Table S1. Characterization of melanoma cell lines

Cell Line	Sex/Age	Primary/ Metastatic	TNM	Stage	BRAF Exon11 Exon15	NRAS Exon1 Exon2
Mel66-CL	44/M	Primary	pT4bN0M0	IIC	V600E/V600E	WT
Mel16-P	46/F	Primary	pT4bN3M1	IV	WT	WT
Mel77-DM	71/M	Primary	pT4bN3M0	IV	V600E	WT
Mel16-MCB	46/F	Metastatic (Cutaneous)	pT4bN3M1	IV	WT	WT
Mel13-SA	62/M	Metastatic (Cutaneous)	pT3bN0M1	IV	WT	Q61R
Mel16-Ldx	46/F	Metastatic (Lymph node)	pT4bN3M1	IV	WT	WT

Table S2. List of primer pairs used for RT-PCR for gene expression analysis

Target mRNA	Primer Forward	Primer Reverse
bFGF	5′-CTGGCTTCTAAATGTGTTACGGA-3′	5′-GCCCAGGTCCTGTTTTGGAT-3′
END-1	5′-CTACTTCTGCCACCTGGACATC-3′	5′-TCACGGTCTGTTGCCTTTGTGG-3′
END-3	5′-CTTTTGCACCCAAACTCTGGACG-3′	5′-ATGGTGGAGGTCTAAAGCCTGC-3′
HGF	5′-CGCTGACAATACTATGAATGACAC-3′	5′-GCTCGTGAGGATACTGAGAATC-3′
IGF-1	5′-ATCAGCAGTCTTCCAACCCA-3′	5′-TGGTGTGCATCTTCACCTTCA-3′
SCF	5′-AAGAGGATAATGAGATAAGTATGTTG-3′	5′-TTACCAGCCAATGTACGAAAGT-3′
TGFβ	5′-GCCCTGGACACCAACTATTG-3′	5′-CGTGTCCAGGCTCCAAATG-3′
VEGF	5′-GTTGACCTTCCTCCATCC-3′	5′-TTCTCTGCCTCCACAATG-3′
IL-1α	5′-CGCCAATGACTCAGAGGAAGA-3′	5′-AGGGCGTCATTCAGGATGAA-3′
IL-1β	5′-CTGAGCTCGCCAGTGAAATG-3′	5′-TTTAGGGCCATCAGCTTCAAA-3′
IL-6	5′-AGCCACTCACCTCTTCAGAACG-3′	5′-GGTTCAGGTTGTTTTCTGCCAG-3′
IL-8	5′-CTTGGCAGCCTTCCTGATTTC-3′	5′-TTCTGTGTTGGCGCAGTGTG-3′
IL-17	5′-CCTTGGAATCTCCACCGCAA-3′	5′-GGTAGTCCACGTTCCCATCA-3′
CXCL-1	5′-CCCAAACCGAAGTCATAGCCA-3′	5′-TTCTTAACTATGGGGGATGCAG-3′
CXCL-9	5-′ATTGGAGTGCAAGGAACCCC-3′	5′-TAGTCCCTTGGTTGGTGCTG-3′
CXCL-10	5′-AGCAGAGGAACCTCCAGTCT-3′	5′-AGGTACTCCTTGAATGCCACT-3′
CXCL-12	5′-CCCGGCTGAAGAACAACAAC-3′	5′-TCTCACATCTTGAACCTCTTGT-3′
CXCL-16	5′-CCTATGTGCTGTGCAAGAGGAG-3′	5′-CTGGGCAACATAGAGTCCGTCT-3′
MMP-1	5′-CTGGCCACAACTGCCAAATG-3′	5′-CTGTCCCTGAACAGCCCAGTACTTA 3′
MMP-2	5′-ACAAAGAGTTGGCAGTGCAATA-3′	5′-GCACAAACAGGTTGCAGCTC-3′
MMP-3	5′-CACAGACCTGACTCGGTTCC-3′	5′-GAGTCAGGGGGAGGTCCATA-3′
MMP-7	5′-TGGTCACCTACAGGATCGTA-3′	5′-GGGATCTCTTTGCCCCACAT-3′
MMP-9	5′-GACGATGACGAGTTGTGGTCC-3′	5′-GGCCCTCGAAGATGAAGGGG 3′
MMP-12	5′-GATGCTGTCACTACCGTGGGAA-3′	5′-CAATGCCAGATGGCAAGGTTGG-3′
MMP-13	5′-CAGTTTGCAGAGCGCTACCT-3′	5′-TTCTCGGAGCCTCTCAGTCA-3′
MMP-19	5′-GGCTTCCTACTCCCCATGAC-3′	5′-GTGACAGGTAGTCCACAGGC-3′
TIMP-2	5′-GTTTATCTACACGGCCCCCT-3′	5′-TCGGCCTTTCCTGCAATGAG 3′
E-cadherin	5′-GCCTCCTGAAAAGAGAGTGGAAG-3′	5′-TGGCAGTGTCTCTCCAAATCCG-3′
GPNMB	5′-GTGCTCAATGGAACCTTCAGCC-3′	5′-AGGAATCCTACTCAGCTCCAGG-3′
N-cadherin	5′-CCTCCAGAGTTTACTGCCATGAC-3′	5′-GTAGGATCTCCGCCACTGATTC-3′
B-actin	5′-GACAGGATGCAGAAGGAGATTACT-3′	5′-TGATCCACATCTGCTGGAAGGT-3′
